# Supplementary material for: Serelaxin Alleviates Fibrosis in Thyroid-Associated Ophthalmopathy via the Notch Pathway
Source: Int J Mol Sci. 2023 May 6;24(9):8356. doi: 10.3390/ijms24098356 (PMC10179109; doi:10.3390/ijms24098356)
Supplement: Supplementary file 1 [file ijms-24-08356-s001.zip › supplementary.pdf]

Supplementary Table S1. Primer sequences of genes for qRT-PCR.

Genes	Sequences (5'-3')
COL1A1	F: AAAGATGGACTCAACGGTCTC
	R: CATCGTGAGCCTTCTCTTGAG
MMP2	F: TGACTTTCTTGGATCGGGTCG
	R: AAGCACCACATCAGATGACT
FOS	F: CAGACTACGAGGCGTCATCC
	R: TCTGCGGGTGAGTGGTAGTA
GNG4	F: ACCCACCGTGGAAGCTGAAG
	R: CCCAAGCAAGGGTCCAGGTA
CREB5	F: CCCTGCCCAACCCTACAATG
	R: GGACCTTGCATCCCCATGAT
α-SMA	F: GGGACTAAGACGGGAATCCT
	R: TGTCCCATTCCCACCATCAC
FN1	F: ACAAGCATGTCTCTCTGCCAA
	R: GCAATGTGCAGCCCTCATTT
IL-6	F: CACTGGTCTTTTGGAGTTTGAG
	R: GGACTTTTGTACTCATCTGCAC
IL-8	F: CCACCGGAAGGAACCATCTC
	R: GGGGTGGAAAGGTTTGGAGT
CXCL1	F: TTCACAGTGTGTGGTCAACAT
	R: AAGCCCCTTTGTTCTAAGCCA
CXCL2	F: AGTGTGTGGTCAACATTTCTCA
	R: GCTCTAACACAGAGGGAAACAC
CCL2	F: CCTTCATTCCCCAAGGGCTC
	R: CTTCTTTGGGACACTTGCTGC
CCL8	F: ATGCTGAAGCTCACACCCTTGCCC
	R: CAGATGCTTCATGGAATCCCTGACC
GAPDH	F: TTGCCATCAATGACCCCTT
	R: CGCCCCACTTGATTTTGGA
