# Supplementary figures and images for: Serelaxin Alleviates Fibrosis in Thyroid-Associated Ophthalmopathy via the Notch Pathway
Source: Int J Mol Sci. 2023 May 6;24(9):8356. doi: 10.3390/ijms24098356 (PMC10179109; doi:10.3390/ijms24098356)

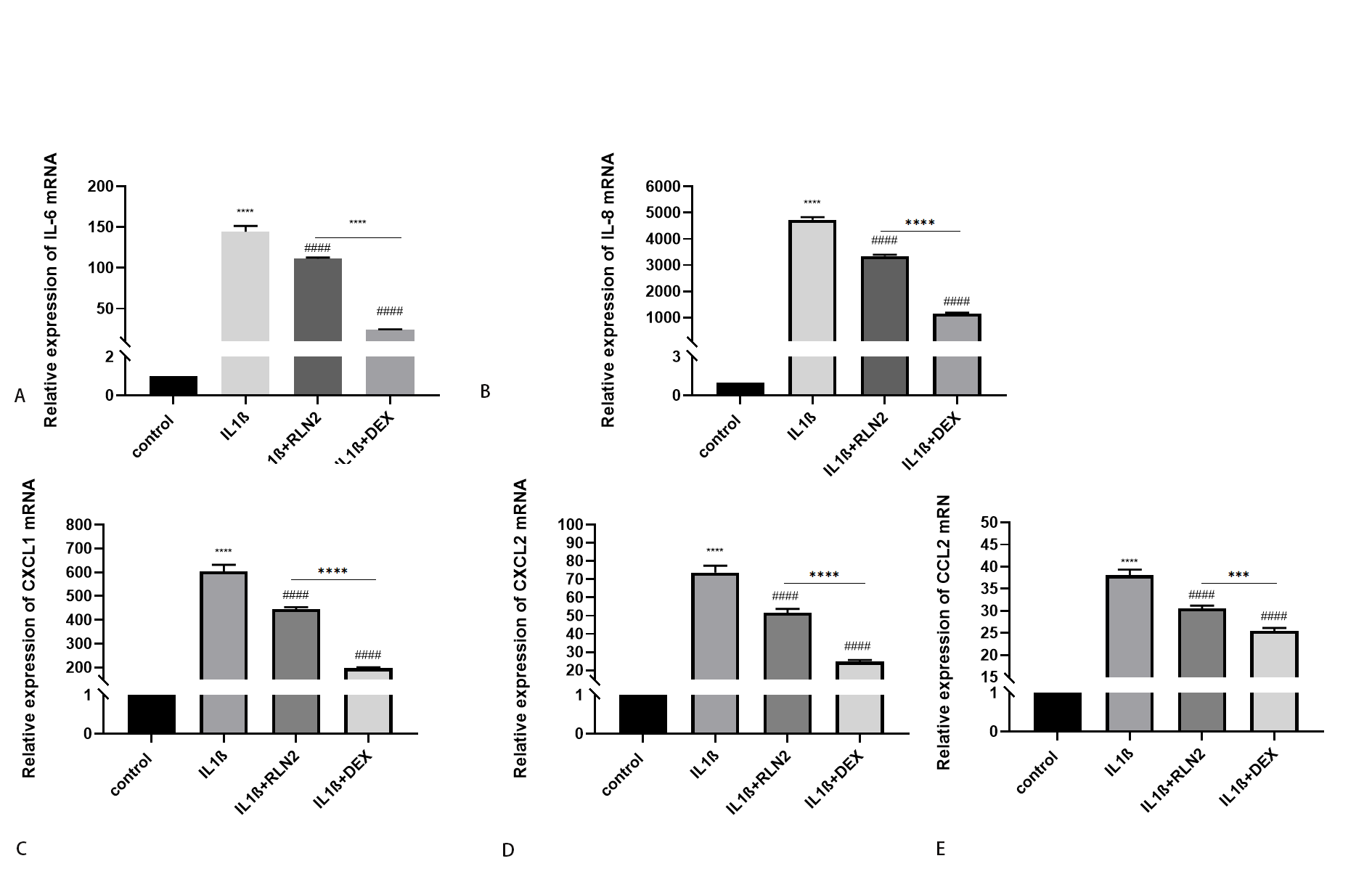

Supplement: Supplementary file 1 [file ijms-24-08356-s001.zip › sunsupplementary Fig2.tif]

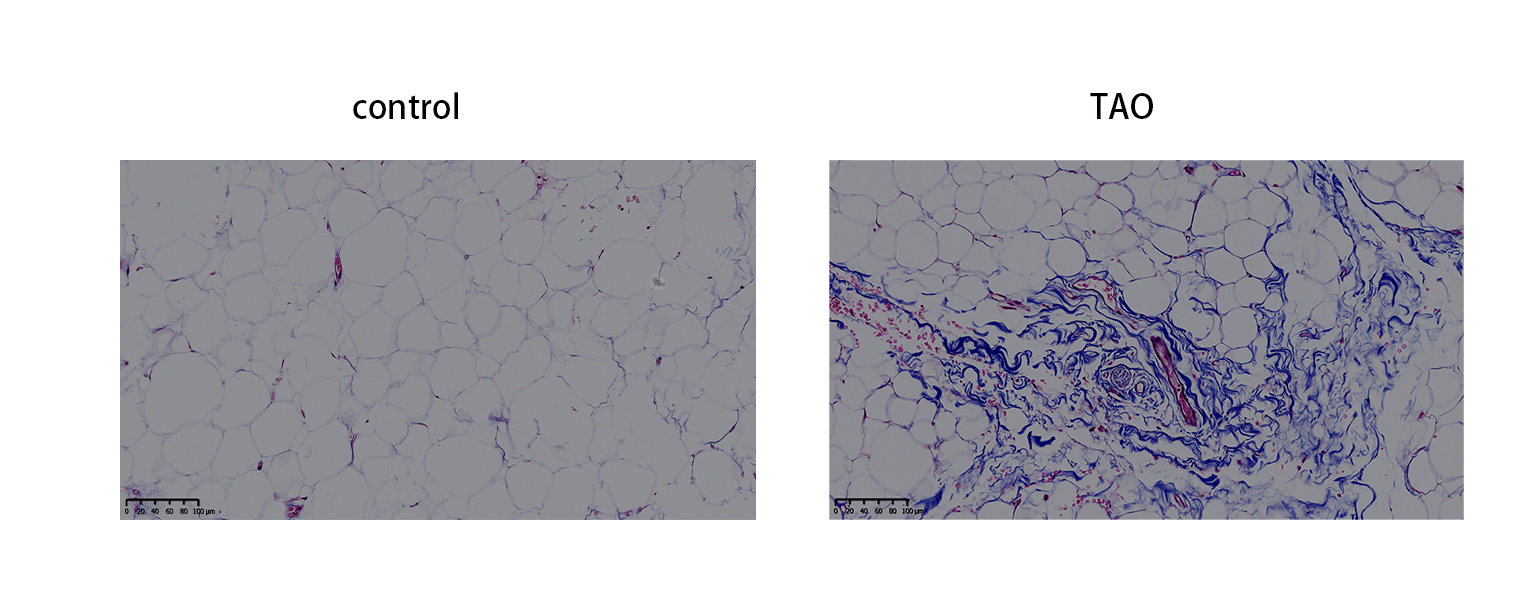

Supplement: Supplementary file 1 [file ijms-24-08356-s001.zip › supplementary Fig1.png]
